# Evaluation of Disulfiram Drug Combinations and Identification of Other More Effective Combinations against Stationary Phase *Borrelia burgdorferi*

**DOI:** 10.3390/antibiotics9090542

**Published:** 2020-08-26

**Authors:** Hector S. Alvarez-Manzo, Yumin Zhang, Wanliang Shi, Ying Zhang

**Affiliations:** Department of Molecular microbiology and Immunology, Bloomberg School of Public Health, Johns Hopkins University, Baltimore, MD 21205, USA; halvare3@jhu.edu (H.S.A.-M.); yzhan424@jhmi.edu (Y.Z.); wshi3@jhu.edu (W.S.)

**Keywords:** Lyme disease, PTLDS, *Borrelia burgdorferi*, persistent infection, disulfiram, nitroxoline

## Abstract

Lyme disease, caused by *Borrelia burgdorferi*, is the most common vector-borne disease in USA, and 10–20% of patients will develop persistent symptoms despite treatment (“post-treatment Lyme disease syndrome”). *B. burgdorferi* persisters, which are not killed by the current antibiotics for Lyme disease, are considered one possible cause. Disulfiram has shown to be active against *B. burgdorferi*, but its activity against persistent forms is not well characterized. We assessed disulfiram as single drug and in combinations against stationary-phase *B. burgdorferi* culture enriched with persisters. Disulfiram was not very effective in the drug exposure experiment (survival rate (SR) 46.3%) or in combinations. Clarithromycin (SR 41.1%) and nitroxoline (SR 37.5%) were equally effective when compared to the current Lyme antibiotic cefuroxime (SR 36.8%) and more active than disulfiram. Cefuroxime + clarithromycin (SR 25.9%) and cefuroxime + nitroxoline (SR 27.5%) were significantly more active than cefuroxime + disulfiram (SR 41.7%). When replacing disulfiram with clarithromycin or nitroxoline in three-drug combinations, bacterial viability decreased significantly and subculture studies showed that combinations with these two drugs (cefuroxime + clarithromycin/nitroxoline + furazolidone/nitazoxanide) inhibited the regrowth, while disulfiram combinations did not (cefuroxime + disulfiram + furazolidone/nitazoxanide). Thus, clarithromycin and nitroxoline should be further assessed to determine their role as potential treatment alternatives in the future.

## 1. Introduction

Lyme disease (LD) is a tick-borne infection caused by *Borrelia burgdorferi* sensu lato complex. Although 30,000 cases are reported to the Centers for Disease Control and Prevention annually, it is estimated that the real number of cases per year in the USA is at least 300,000, making it the most common vector-borne infection in the country [1]. Treatment of LD is carried out with a 2–4 week course of antibiotic treatment with doxycycline, or amoxicillin, or cefuroxime. Despite treatment, 10–20% of patients with LD diagnosis will develop persistent symptoms such as fatigue, musculoskeletal pain [2], arthralgia, and neurological and neuropsychological impairment, including verbal and visual memory loss [3]. Symptoms that persist longer than 6 months despite treatment will usually prevail over time [4] and consolidate as sequela, with impairment in daily life activities and in health-related quality of life [3], a condition called post-treatment Lyme disease syndrome (PTLDS) [2,5].

While the real cause of PTLDS is unclear, several theories have been put forward to explain the cause of PTLDS. The presence of antigenic debris after antimicrobial treatment could trigger the immune response, causing some of the symptoms in PTLDS [6]. Other explanations include an autoimmune response following infection and the presence of a persistent infection that is difficult to identify by the current diagnostic methods [5]. There have been in vivo studies in rodents, dogs, and non-human primates that have shown the persistence of *B. burgdorferi* by PCR after antimicrobial treatment [7,8,9]. Moreover, in one study in humans, xenodiagnosis of a patient with PTLDS was found to have positive *B. burgdorferi* DNA in the xenodiagnostic tick, although the organism could not be cultured [10].

It has been shown that *B. burgdorferi* in a stationary phase culture (7–10 days old) is enriched with persistent forms, which include the microcolony and the round-body forms [11]. Although these forms have not been proven to be clinically significant, in vitro studies have shown the importance of these forms as antibiotic-tolerant persisters [11]. The relevance of these persister forms in causing more severe and persistent disease that is more difficult to cure with the current Lyme antibiotics was recently shown in a mouse model [12]. In addition, the conventional antibiotics used to treat patients with LD have failed to eradicate the persistent forms of *B. burgdorferi* in in vitro experiments, and only the persister drug daptomycin plus doxycycline and a cephalosporin in a three-drug combination has been able to eradicate the aggregated persistent forms [13,14,15,16,17,18]. More importantly, the daptomycin three-drug combination was also able to eradicate *Borrelia* persisters in a mouse model that are not killed by the current Lyme antibiotics [12]. Therefore, the above findings validate the relevance of the in vitro drug combination study. However, because daptomycin is an expensive intravenous antibiotic, there is interest to identify other oral persister drugs that can replace daptomycin. Thus, it is important to identify other drugs that are active against persistent forms of the disease, both as single agents and in combination with other drugs.

Recently, it has been reported that disulfiram (DSF) is active against *B. burgdorferi* [19]. This drug is a synthetic, organosulfur-based drug that is used to induce abstinence in patients who suffer alcoholism. The active metabolite of DSF is diethyldithiocarbamate (DETC), and this molecule binds to aldehyde dehydrogenase (ALDH), causing inhibition of the enzyme and elevated levels of acetaldehyde, which is a chemical responsible for the “hangover” after alcohol consumption [20]. A drug screening conducted in 2016 [19] identified DSF as an effective drug against the growing form of *B. burgdorferi*, with a minimum inhibitory concentration (MIC) of 0.625 µM (equivalent to 0.18 µg/mL) and a minimum bactericidal concentration (MBC) of 1.25 µM (equivalent to 0.38 µg/mL). In the same study, DSF at a concentration of 1.25 µM inhibited 99.8% of the borrelial growth in a stationary phase culture when compared with the untreated control. The same research group further evaluated the efficacy of DSF in a stationary-phase culture in vitro, as well as in a LD mouse model [21]. The experiments showed inhibition of *B. burgdorferi* growth of approximately 90% when adding DSF at 1.5 µg/mL to a stationary-phase culture. In the case of the animal protocol, most of the mice cleared the infection in most of the tissues tested by qPCR after 21 or 28 days of *B. burgdorferi* inoculation when treated intraperitoneally with DSF at 75 mg/kg for 5 days. However, the authors claim that clearance of infection of mice at a lower dose (10 mg/kg) of DSF was not possible. Additionally, a case report was published in which three patients with relapsing neurological LD symptoms improved their health conditions by a sole DSF therapy for 6 to 18 weeks, depending on the case [22]. Moreover, DETC has shown to be an active molecule against growing and non-growing forms of *Mycobacterium tuberculosis* in in vitro studies [23]. In addition, DSF was shown to disrupt the biofilm of *Pseudomonas aeruginosa* in vitro [24]. However, drug combination studies with DSF in a *B. burgdorferi* stationary-phase culture have not been performed.

In this study, we evaluated DSF in comparison with other *Borrelia* persister drugs and then in combination with other antibiotics in two- and three-drug combinations. The antibiotics tested were either antibiotics conventionally used for the treatment of LD, or antimicrobial agents with a strong activity against *B. burgdorferi* persisters [11]. We used the optimized SYBR Green I/Propidium Iodide (SYBR Green I/PI) assay previously described to assess bacterial viability [25] and the most promising drug combinations were evaluated by a subculture study to confirm their ability to completely eradicate persisters without regrowth.

## 2. Results

### 2.1. MIC Testing and Relative Activity of DSF at 50 µM

In this study, we evaluated the activity of DSF as a single drug, as well as in two- and three-drug combinations. The MIC for DSF in our study was of 0.3 µg/mL, while cefuroxime (CefU) and doxycycline (Doxy) showed lower MICs at 0.15 µg/mL and 0.08 µg/mL, respectively (Table 1). Moreover, when comparing DSF with other drugs at a 50 µM concentration for stationary phase *B. burgdorferi*, we found a survival rate of 39.4%, slightly above the CefU value of 35.4%. However, it still was not as effective in the eradication of *B. burgdorferi* as other drugs with higher anti-persister activity such as clarithromycin (Clari; survival rate 14.3%), furazolidone (FZD; survival rate 5.0%), nitroxoline (NTX; survival rate 1.5%), and nitazoxanide (NTZ; survival rate 0.8%), among others (Table S1, Figure S1).

### 2.2. Activity against Stationary-Phase Culture at Standard Dose of 5 µg/mL and Cmax Concentration

DSF tested as a single drug at 5 µg/mL was shown once again to be similarly as effective as the standard treatment with CefU (Table S2), with a survival rate of 34.1% and 36.0%, respectively. Furthermore, the best drugs at this concentration were cryptolepine (Cry) and NTX, with survival rates of 32.1% and 32.6%, respectively. When adding a second drug, Cry and rifabutin (Ribu) in combination with DSF showed good activity, with survival rate values below 20%. Moreover, two-drug combinations with DSF were in general more effective than the CefU + Doxy combination (which served as the two-drug control).

Combinations highlighted in Table S2 were further assessed as three-drug combinations at 5 µg/mL concentration (Table S3) by adding a drug with anti-persister activity.

In this further testing, no combination with CefU + Doxy managed to keep bacterial viability at 10% or below, but CefU + Doxy + Cry was the best drug combination, with a value of 13.1%. In comparison, CefU + DSF + Cry showed a survival rate of 1.4%. The same pattern was observed with other three-drug combinations that included DSF. We set up 73 three-drug combinations at 5 µg/mL that included DSF and identified 17 with survival rates at 10% or below (highlighted in Table S3). These were further assessed at Cmax concentrations to simulate the peak concentration of drugs in serum after oral ingestion (Table 2).

**Figure 1:**
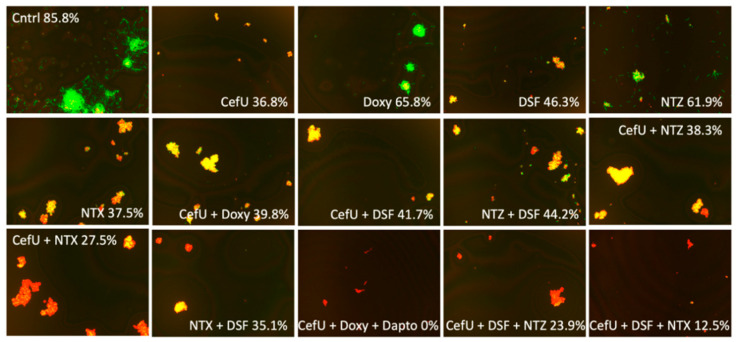
Effect of single drugs, and two-drug and three-drug combinations of interest at Cmax concentration. Abbreviations: untreated control (Cntrl), cefuroxime (CefU), daptomycin (Dapto), doxycycline (Doxy), disulfiram (DSF), doxycycline (Doxy), nitazoxanide (NTZ), nitroxoline (NTX).

We assessed 17 possible drug combinations that maintained their survival rate values at 10% or below at the standard dose of 5 µg/mL. Along with these, we also evaluated single drugs and two-drug combinations in order to have a better understanding of the synergy of the different combinations at Cmax concentrations (Figure 1). CefU showed the best result with a survival rate of 36.8%, followed by NTX and Clari with values of 37.5% and 41.1%, respectively. In comparison, Cry survival rate adjusted at Cmax concentration did not maintain good activity (survival rate of 63.3%), and DSF (survival rate of 46.3%) showed a difference of almost 10% when compared to CefU. When assessing the drugs in two-drug combinations, CefU + Clari and CefU + NTX were the most effective, with survival rates of 25.9% and 27.5%, respectively. Furthermore, DSF two-drug combinations were less active than CefU two-drug combinations (with the exception of DSF + FZD). Finally, we evaluated DSF three-drug combinations. We found only one potential combination that kept bacterial viability at 12.5%—CefU + DSF + NTX. The next best combinations were CefU + DSF + NTZ (survival rate of 23.9%) and CefU + DSF + FZD (survival rate 25.1%). In addition, we took the three-drug combination CefU + Doxy + daptomycin (Dapto) (survival rate 0%) at Cmax as our positive control on the basis of our previous work. It proved to indeed be the best drug combination against a *B. burgdorferi* stationary-phase culture since the survival rate was in all repetitions maintained at 0%.

**Figure 2:**
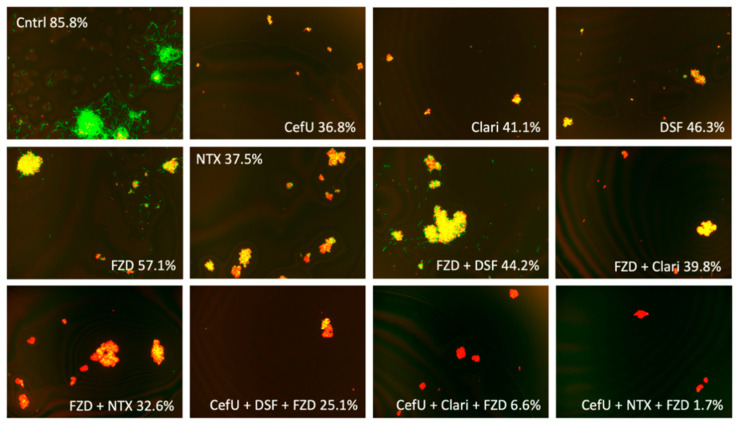
Effect at Cmax concentration for combinations with DSF, Clari, and NTX. Abbreviations: untreated control (Cntrl), cefuroxime (CefU), clarithromycin (Clari), disulfiram (DSF), furazolidone (FZD), nitroxoline (NTX).

We chose two DSF three-drug combinations at Cmax and replaced DSF with Clari or NTX in order to evaluate the direct effect of each drug on the survival rate of the bacteria in comparison to DSF (Table 3, Figure 2). The combinations chosen were CefU + DSF + FZD and CefU + DSF + NTZ. Thus, the new combinations after replacing DSF with Clari or NTX were the following: CefU + Clari/NTX + FZD and CefU + Clari/NTX + NTZ. After performing the drug-exposure experiment in a stationary-phase culture, we found that the survival rate for CefU + Clari + FZD and CefU + NTX + FZD (survival rates of 6.6% and 1.7%, respectively) were statistically significantly better than the combinations with DSF (survival rate of 25.1%). Similarly, CefU + Clari + NTZ and CefU + NTX + NTZ (survival rates of 5.0% and 11.0%, respectively) proved to be better combinations when compared to the DSF combination (survival rate of 23.9%); however, only the three-drug combination with Clari was statistically significant (Table 3).

We also performed a statistical analysis in order to determine the activity of the single drugs evaluated at Cmax (Table 2). NTX (survival rate of 37.5%) and Clari (survival rate of 41.1%) as single drugs were found to be equivalent to CefU (survival rate of 36.8%). Moreover, CefU + Clari and CefU + NTX (survival rates of 25.9% and 27.5%, respectively) were statistically significant when compared to the current Lyme antibiotics CefU + Doxy two-drug control (survival rate of 39.8%). Three-drug combinations with DSF at Cmax (Table 2) failed to keep bacterial viability low in a stationary-phase culture, wherein CefU + DSF + NTX was the only three-drug combination at Cmax that remained effective against a stationary-phase *B. burgdorferi* culture with a survival rate of 12.5% (Figure 1).

### 2.3. Subculture Study

**Figure 3:**
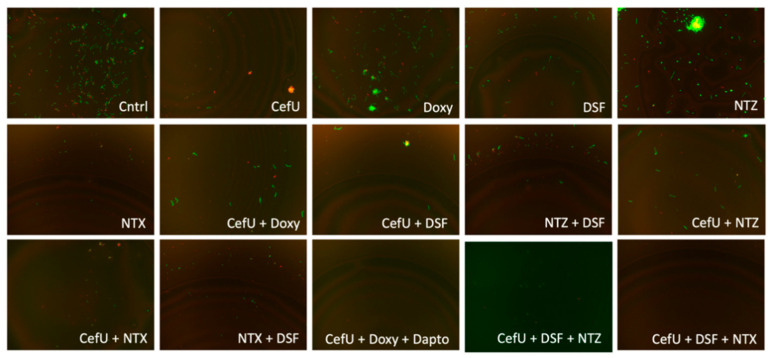
Subculture for single drugs, as well as for two-drug and three-drug combinations. Abbreviations: untreated control (Cntrl), cefuroxime (CefU), daptomycin (Dapto), doxycycline (Doxy), disulfiram (DSF), doxycycline (Doxy), nitazoxanide (NTZ), nitroxoline (NTX).

**Figure 4:**
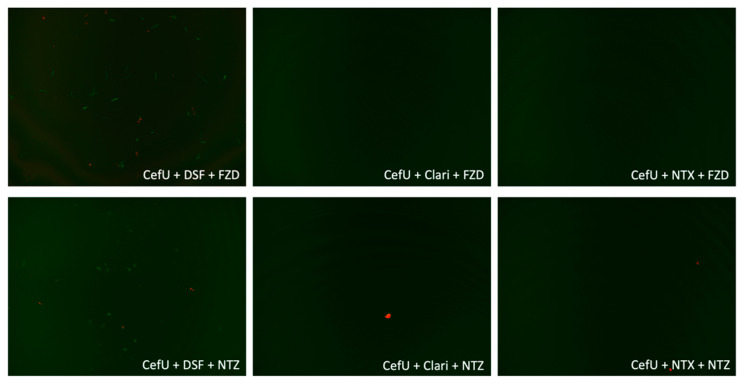
Subculture for comparative combinations with DSF, Clari, and NTX. Abbreviations: untreated control (Cntrl), cefuroxime (CefU), clarithromycin (Clari), disulfiram (DSF), furazolidone (FZD), nitroxoline (NTX).

We assessed CefU + DSF + NTX, CefU + DSF/Clari/NTX + NTZ, and CefU + DSF/Clari/NTX + FZD in a subculture study, along with the three-drug control CefU + Doxy + Dapto, which showed complete eradication of stationary phase *B. burgdorferi* (0% viability) and has been previously described as the best combination effective for the eradication of *B. burgdorferi* in vitro [16]. After 3 weeks, CefU + DSF + NTX, along with the three-drug control CefU + Doxy + Dapto, showed no regrowth (Table 4, Figure 3). However, CefU + DSF + NTZ and CefU + DSF + FZD were not able to eradicate bacteria and regrew after 21 days of subculture. The four combinations with Clari and NTX (CefU + Clari + NTZ, CefU + NTX + NTZ, CefU + Clari + FZD, CefU + NTX + FZD) at Cmax concentrations prevented the regrowth of *B. burgdorferi* in the subculture experiment after 3 weeks, while combinations with DSF (CefU + DSF + NTZ and CefU + DSF + FZD) regrew (Table 4, Figure 4).

## 3. Discussion

In this study, we assessed the performance of DSF in different experiments at different concentrations as a single drug and in two- and three-drug combinations in comparison with other promising drug candidates in order to determine the effectiveness of this drug in the eradication of *B. burgdorferi*. In the MIC test, the DSF MIC-value of 0.3 µg/mL was consistent with the previous study, in which the MIC-value for DSF was of 0.18 µg/mL [19]. Since the Cmax value for DSF was 0.4 µg/mL, this result suggests that DSF could be useful against the growing form of *B. burgdorferi*. However, we were more interested in the performance of DSF activity against a stationary phase culture enriched with persisters that was based on the persister drug study with daptomycin in vitro [16] and in vivo [12]. We therefore evaluated the relative activity of DSF at 50 µM compared with other drugs against stationary-phase *B. burgdorferi*. In this case, DSF was shown to be almost equally effective as CefU, with a survival rate of 39.4% (CefU survival rate of 35.4%). However, many of the drugs tested at this high concentration showed a survival rate below 30% (artemisinin (Arte), azithromycin (Azi), Clari, clofazimine (CFZ), Cry, erythromycin (Ery), FZD, linezolid (LNZ), NTZ, NTX, and Ribu), suggesting that some of these drugs might be more effective against the persister forms of *B. burgdorferi* than DSF and CefU at 50 µM. Moreover, CefU and Amoxi survival rate values were consistent with previous studies carried out by our group [17]. In the specific case of Clari 50 µM (survival rate of 14.3%), our results in this study indicated that it was more active than that in our previous study [11]. We think the main reason for this discrepancy is that in the previous screening, a bacterial counting chamber was used in order to determine bacterial viability, while in the present study, we used ImageJ software to measure the green and red fluorescence bacteria in both aggregated and planktonic forms. We believe a bacterial counting chamber, which tends to count only planktonic cells, is partially accurate to determine the amount of live or dead bacterial cells, since *B. burgdorferi* aggregated microcolonies in stationary-phase cultures may not be counted. Nevertheless, there is overall agreement with the results with the current Lyme antibiotics and known persister drug combinations with daptomycin, which showed better activity than the current Lyme antibiotics [16].

When testing the antibiotics in a stationary phase culture at 5 µg/mL, most of the two-drug combinations with DSF showed a better eradication activity in the 7-day-old stationary-phase culture when compared to the CefU two-drug control. In the case of the three-drug combinations, even though no combination achieved a result of 0% like the three-drug control with CefU + Doxy + Dapto, 17 DSF combinations achieved good results of 10% or less viability (Table S3). The most active drugs in a three-drug combination with DSF were Clari, Cry, FZD, LNZ, NTZ, NTX, and Ribu. In comparison, no CefU + Doxy three-drug combination managed to maintain a survival rate of 10% or less, and the most effective combinations were CefU + Doxy + Cry and CefU + Doxy + NTX, with survival rates of 13.1% and 17.5%, respectively. In addition, we evaluated single drugs as well as two-drug and three-drug combinations at Cmax in a subculture study and found that CefU + DSF + NTX is an effective combination for the eradication of *B. burgdorferi* in a stationary phase culture. However, CefU + DSF + NTZ and CefU + DSF + FZD did not work as well. Due to significant adverse reactions related to the DSF uptake [26], especially neurological (i.e., neuropathies, headaches) and psychiatric symptoms (i.e., anxiety, suicidal thoughts, concentration difficulties), we focused on other drugs that can replace DSF and are still able to eradicate *B. burgdorferi* persisters. On the basis of the experiments we performed, we decided to replace DSF with Clari or NTX in two three-drug combinations: CefU + DSF + FZD and CefU + DSF + NTZ. By doing this, we sought to compare the effect of Clari and NTX at Cmax concentration in three-drug combinations and directly compare these with the results from the combinations with DSF. In both cases, the replacement with Clari or NTX showed a statistically significantly lower survival rate than combinations with DSF (Table 3). Moreover, Clari or NTX combinations (CefU + Clari + NTZ, CefU + NTX + NTZ, CefU + Clari + FZD, CefU + NTX + FZD) did not regrow after 3 weeks, while DSF combinations did not eradicate *B. burgdorferi* after the same period of time. This result showed that even if we found one three-drug combination with DSF that completely eradicated *B. burgdorferi*, Clari and NTX seem to be, at least in vitro, better alternatives.

There have been previous case reports of patients diagnosed with PTLDS and treated with DSF [22,26]. Patients in these studies reported improvement in their health condition after treatment with DSF. However, empirical treatment without the proper in vitro and in vivo validation is difficult to assess since physicians cannot be sure that DSF causes the clinical improvement due to confounding factors. In addition, in both articles, the indication of treatment with DSF was based on the results of an in vitro study [19]. In a study published in 2016, Pothineni V.R. and colleagues demonstrated with a Bac-Titer-Glo assay that a dose of 1.25 µM (equivalent to 0.38 µg/mL) would inhibit bacterial growth by 99.8%. However, the authors also stated that their assay might not be sensitive to bacteria that are metabolically inactive due to persistence, and although this has not been tested, we believe the difference between the results reported in that study and our results might be due to the different assays used. In our study, we used the SYBR Green I/PI assay to assess bacterial viability after drug exposure. The SYBR Green I/PI assay has been previously validated and has been shown to be the most sensitive method to assess bacterial viability in *B. burgdorferi* in both the planktonic and microcolony form [27], which are highly prevalent in stationary-phase cultures. In our study, DSF as a single drug at Cmax concentration (0.38 µg/mL) inhibited *B. burgdorferi* in a 7-day-old stationary phase culture by 53.7% (survival rate of 46.3%). Moreover, two- and three-drug combinations grew in the subculture studies (except for CefU + DSF + NTX), but when replacing DSF with other drugs such as Clari or NTX, subcultures remained negative. These data suggest that DSF activity against persister forms is not as effective as previously thought, but gives us the hint that other antibiotics (i.e., nitroxoline) could be better options for killing the non-growing persister forms of *B. burgdorferi*. In addition, as far as we know, no drug combinations with DSF or subculture studies with DSF have been previously published. We think that in order to repurpose DSF as a possible treatment in humans, treatment of mice infected with *B. burgdorferi* should be performed. However, in order to do so, drug combinations have to be assessed in in vitro experiments to select those combinations with DSF (or other antibiotics) that could potentially work in an animal model. In this study we did not address expression of genes, molecular targets, or resistance/persistence mechanisms. However, we believe it was important to perform these experiments through identifying potentially more effective drug candidates or combinations than DSF, since empirical treatment with disulfiram is still taking place and may be doing more harm due to toxicity than it is in improving the health outcome of patients with PTLDS [26]. Thus, our results are relevant for questioning the previous hypothesis of the activity of DSF against persister forms of *B. burgdorferi* [19].

The MIC for Clari was of 0.04 µg/mL, which is consistent with a previous research paper published [28]. Clari has been used for at least 12 months for the treatment of persistent *Mycobacterium avium* complex infections [29]. This suggests that Clari might have some effect on persistent forms of bacteria and in order to determine it, further analysis with Clari should be performed. In the case of NTX, it is an antibiotic used for the treatment of urinary tract infections. However, it has also been repurposed for the treatment of biofilm infections and some bacterial infections due to multidrug resistant Enterobacteriaceae [30]. The survival rate of NTX in our study, in addition to the fact that this antibiotic is currently being repurposed for other bacterial infections, suggests that NTX could be an alternative candidate for consideration of treatment of LD, if further in vitro and in vivo activity is validated. Nevertheless, its combination with other drugs against *B. burgdorferi* still has to be assessed in further studies.

Another interesting drug that we assessed is Cry. Cry is an alkaloid molecule extracted from the plant *Cryptolepis sanguinolenta*. In a previous study, it has shown to be very active in the eradication of *B. burgdorferi* in a stationary-phase culture [31]. Our results at 5 µg/mL suggest that Cry is very active in combination with other antibiotics. Some studies in Africa report the use of Cry in teabags for the treatment of malaria [32]. However, an oral formulation of Cry is not available, and hence its Cmax concentration in humans is not available. The Cmax concentration we used was taken from a pharmacokinetic study in mice. We realize that the Cmax concentration we used in this study for Cry was extremely low (0.024 µg/mL) when compared to all other antibiotics tested and that the study from which we took the Cmax value was conducted with only two mice [33]. We think further pharmacokinetic studies of Cry should be performed in order to evaluate a more exact Cmax value, evaluate the potential toxicity of this compound, and determine its potential use for LD.

We found that Clari and NTX at Cmax concentrations were as active as CefU and more active than DSF. Furthermore, two-drug combinations at Cmax with CefU + Clari/NTX were in general equally active to most of the three-drug combinations with DSF. The second best three-drug combination with DSF, CefU + DSF + NTZ (survival rate of 23.9%), showed a difference of only 2% and a reduction in bacterial viability of only 8% when compared to CefU + Clari (survival rate of 25.9%). Thus, it is reasonable to believe that by adding a third drug to these two-drug combinations (CefU + Clari and CefU + NTX) it will result in better eradication rates than three-drug combinations with DSF. Lastly, only one three-drug combination with DSF showed an inhibition of regrowth in the subculture study. Interestingly, this combination in particular included CefU and NTX, which are the two drugs that showed the best single results at Cmax concentration. It is important to mention that NTX is not a drug available in the USA. Thus, the clinical relevance of this specific drug in the USA is limited. However, it is a drug commercially available in Europe, for example in Germany and other Eastern European countries [34,35], where it is used for the treatment of urinary tract infections [36]. Europe’s prevalence of Lyme disease is actually estimated to be three times higher than the number of cases reported in the USA, with the majority of cases occurring in Germany [37], Austria, Lithuania, Slovenia, and Sweden [0]. Hence, we believe that these results are relevant, since if further investigated with *B. garinii* and *B. afzelii* (the species causing Lyme disease in Europe), nitroxoline could be repurposed for the treatment of LD in these countries. We hypothesize that two- and three-drug NTX combinations with other drugs with anti-persiter activity could eradicate *B. burgdorferi* in a stationary-phase culture at least with the same effectiveness as CefU. Further studies are needed to assess promising NTX combinations in vitro, as well as in LD animal models to support the date obtained. Moreover, it is also possible to assess studies of spirochete growth in cell lines in the presence of antibiotics, as previous studies have reported [38]. Such ex vivo experiments could be potentially useful to determine in a more similar way in vivo conditions, with the advantage of not using an animal model.

## 4. Materials and Methods

### 4.1. Strain, Media, and Culture Techniques

For the culture of *B. burgdorferi* N40 strain, we used Barbour-Stoenner-Kelly-H (BSK-H) medium (HiMedia Laboratories Pvt Ltd., Mumbai, India) supplemented with 6% rabbit serum (Sigma-Aldrich, St. Louis, MO, USA). BSK-H medium was filter-sterilized with 0.2 µM filters and no antibiotics were added. Cultures were incubated in sterile 15 mL tubes (BD Biosciences, San Jose, CA, USA) and placed in a microaerophilic incubator (33 °C, 5% CO_2_). After 7–10 days, once the culture reached the stationary phase, which is equivalent to approximately 107–108 spirochetes per milliliter on the basis of previous studies [11], we transferred 100 µL of the culture to each well of a 96-well plate for the evaluation of drugs (100 µL per well). Plates were sealed and stored in the incubator for 7 days without shaking.

### 4.2. Drug Selection

The following drugs were used in this study: amoxicillin, artemisinin, azithromycin, cefuroxime, clarithromycin, clofazimine, cryptolepine, daptomycin, disulfiram, doxycycline, erythromycin, furazolidone, linezolid, nitazoxanide, nitroxoline, and rifabutin. All drugs were purchased from Sigma-Aldrich (St. Louis, MO, USA) and were dissolved in the proper solvents. The decision to utilize these drugs was based on previous drug screenings [11,14], anti-persister activity of some of them assessed in pilot experiments, and the fact that some of these drugs are normally used for the treatment of LD. In addition, we only evaluated antibiotics that can be orally administered. Drug stock-solutions dissolved in water were filter-sterilized with 0.2 µm filters while drugs dissolved in DMSO were not filtered and the stock solutions were kept at −20 °C.

### 4.3. Microscopy

*B. burgdorferi* cultures were assessed with a BZ-X710 all-in-one fluorescence microscopy (KEYENCE, Itasca, IL, USA). Samples were stained with the SYBR Green I/PI assay and the bacterial viability was assessed by calculating the green/red fluorescence ratio as described previously [25]. A stock solution of SYBR Green I/PI was prepared with 10 µL of SYBR Green I (10,000× stock, Invitrogen) and 30 µL of PI (20 µM, Sigma) into 960 µL of sterile dH_2_O. Then, the stock solution was diluted in a 1:4 ratio with sterile dH_2_O. For this study, 7 µL of the work solution and 14 µL of sample were added to each well for observation under the microscope. Drug combinations were assessed three times and a representative picture of each sample was taken for further analysis. In order to determine the green/red fluorescence, we employed the software ImageJ with the objective to highlight and more easily count bacterial cells stained green or red. We calculated the survival rate by dividing the green fluorescence over green and red fluorescence (Green/(Green + Red)).

### 4.4. Drug Susceptibility Testing

For the MIC testing of the drugs, the microdilution method was employed, and the bacterial growth inhibition was assessed by microscopy after SYBR Green/PI staining assay. The next step was to evaluate the relative activity of each of the drugs, which were evaluated at a 50 µM concentration in a stationary phase culture. For the drug combinations, a final concentration of 5 µg/mL was chosen for the drug testing. This concentration was selected on the basis of approximate means of the Cmax of the drugs tested in this research protocol. All drugs were evaluated as single drugs and two-drug combinations with CefU and DSF in order to contrast DSF activity against *B. burgdorferi* with CefU (current Lyme treatment along with doxycycline). The best two-drug combinations were further tested in combination with a third drug with anti-persister activity. It is important to mention that the three-drug combination CefU + Doxy + Dapto was used as a positive control, since it has shown in our previous studies to be the best drug combination for the eradication of a *B. burgdorferi* stationary-phase culture enriched with persister forms [17]. Combinations that showed a bacterial viability of 10% or less were further studied at Cmax concentration to better simulate the peak concentration in serum after oral ingestion in humans. Cmax concentrations were taken from the literature (Table 5).

### 4.5. Subculture Study

For those combinations that kept a bacterial viability of 10% or less after adjusting to Cmax concentrations, we performed a subculture study in order to ensure bacterial eradication without regrowth. Drug combinations at Cmax concentrations were added to 1 mL of a 7-day-old *B. burgdorferi* and were incubated for 7 days. After treatment, samples were spun down and processed for the subculture study, as previously described [27,52]. Samples were evaluated with the SYBR Green I/PI assay for their visualization under the microscope after 3 weeks in order to assess bacterial regrowth. Single drugs and drug combinations tested in the subculture study were assessed in triplicate.

### 4.6. Statistical Analysis

For the single drugs and two-drug combinations at Cmax concentrations, we performed a total of seven repetitions per sample and carried out a statistical analysis with the GraphPad Prism8 software. Since CefU is the preferred antibiotic for the treatment of LD, we considered it important to establish the activity of single drugs at Cmax concentration compared to CefU. In order to do this, we used the Kruskal–Wallis test to establish statistical differences. The same statistical approach was utilized for the rest of the samples and a *p*-value of less than 0.05 was considered significant.

## 5. Conclusions

We assessed the activity of DSF against a *B. burgdorferi* stationary phase culture in comparison with other drugs. On the basis of our results, DSF was not shown to be as effective as previously thought in the eradication of stationary phase *B. burgdorferi*, either as a single drug when compared to CefU or as in two-drug or three-drug combinations. In addition, due to adverse reactions related to DSF, we were interested in finding alternative drug candidates that are more effective and with less side effects than DSF for the treatment of LD. We found that Clari and NTX were equivalent to CefU, showing similar survival rates at Cmax concentration, and even the two-drug combinations with CefU + Clari/NTX were more effective than the two-drug control with CefU + Doxy. We found one three-drug combination with DSF (CefU + DSF + NTX) that inhibited regrowth of *B. burgdorferi*; however, other combinations did not manage to do so (CefU + DSF + FZD, CefU + DSF + NTZ). Since DSF has significant side effects, we replaced DSF with Clari or NTX in the combinations previously mentioned and were able to obtain drug combinations that prevented regrowth in the subculture experiment. Moreover, results in this article should be further tested in animal experiments in order to establish which combinations could actually eradicate *B. burgdorferi* in vivo. At last, we believe Clari and NTX should be further assessed as single drugs and in combinations in in vitro experiments and in animal models in comparison with other Lyme antibiotics in order to determine their roles as potential drug candidates for a more effective treatment of LD.

## Figures and Tables

**Table 1:** Minimum inhibitory concentration (MIC) values. The MIC experiment was tested with the microdilution method.

Drug	MIC (µg/mL)	Drug	MIC (µg/mL)
Cefuroxime (CefU)	0.15	Daptomycin (Dapto)	>10.0
Doxycycline (Doxy)	0.08	Disulfiram (DSF)	0.3
Amoxicillin (Amoxi)	0.3	Erythromycin (Ery)	<0.01
Artemisinin (Arte)	5.0	Furazolidone (FZD)	5.0
Azithromycin (Azi)	0.6	Linezolid (LNZ)	5.0
Clarithromycin (Clari)	0.04	Nitazoxanide (NTZ)	10.0
Clofazimine (CFZ)	5.0	Nitroxoline (NTX)	1.25
Cryptolepine (Cry)	0.6	Rifabutin (Ribu)	5.0

**Table 2:** *Borrelia burgdorferi* viability (in %) in a 7-day-old stationary phase culture after the drug exposure experiment for 7 days at a Cmax concentration for each drug. Results underlined were further assessed in a subculture experiment (Table 4).

	Cntrl	CefU	Doxy	DSF	Arte	Clari	CFZ	Cry	Ery	FZD	LNZ	NTZ	NTX	Ribu
	85.8	36.8	65.8	46.3	53.6	41.1 ^ns^	65.6	63.3	53.0	57.1	56.8	61.9	37.5 ^ns^	48.4
CefU	–––	–––	39.8	41.7	33.7	25.9 *	35.3	38.4	36.7	45.3	41.3	38.3	27.5 *	37.7
DSF	–––	41.7	46.3	–––	41.0	32.3	44.9	42.8	39.8	37.7	44.7	44.2	35.1	39.1
CefU + Dapto	–––	–––	0.0	–––	–––	–––	–––	–––	–––	–––	–––	–––	–––	–––
Arte + DSF	–––	–––	–––	–––	–––	–––	–––	–––	–––	–––	–––	–––	–––	–––
CefU + DSF	–––	–––	–––	–––	–––	–––	–––	37.4	–––	25.1	–––	23.9	12.5	–––
Clari + DSF	–––	–––	–––	–––	–––	–––	–––	–––	–––	–––	28.0	–––	26.8	–––
CFZ + DSF	–––	–––	–––	–––	–––	–––	–––	–––	–––	38.6	–––	–––	–––	29.2
Cry + DSF	–––	–––	–––	–––	–––	–––	–––	–––	36.2	–––	–––	33.3	–––	32.6
FZD + DSF	–––	–––	–––	–––	–––	–––	–––	–––	–––	–––	28.1	32.2	24.0	–––
LNZ + DSF	–––	–––	–––	–––	–––	–––	–––	–––	–––	–––	–––	29.6	25.5	–––
NTZ + DSF	–––	–––	–––	–––	–––	–––	–––	–––	–––	–––	–––	–––	–––	30.0

^ns^ Clari and NTX showed no statistical significance when compared to CefU. * Clari + CefU and Clari + NTX showed statistical significance when compared to CefU + Doxy. A crossed line means values for these combinations were not determined. Abbreviations: Untreated control (Cntrl), artemisinin (Arte), cefuroxime (CefU), clarithromycin (Clari), clofazimine (CFZ), cryptolepine (Cry), daptomycin (Dapto), disulfiram (DSF), doxycycline (Doxy), erythromycin (Ery), furazolidone (FZD), linezolid (LNZ), nitazoxanide (NTZ), nitroxoline (NTX), and rifabutin (Ribu).

**Table 3:** Drug-exposure experiment for combinations with DSF, Clari, and NTX of a 7-day-old stationary phase culture after a 7-day treatment. *B. burgdorferi* viability (in %) in a 7-day-old stationary-phase culture after the drug exposure experiment at a Cmax concentration for each drug. Results underlined are the direct comparison replacing DSF with Clari or NTX.

Drug	Cntrl	DSF	Clari	NTX
–––	85.8	46.3	41.1	37.5
CefU	36.8	41.7	25.9	27.5
FZD	57.1	37.7	34.2	21.4
NTZ	61.9	44.2	39.8	32.6
CefU + FZD	45.3	**25.1 **	6.6 *	1.7 *
CefU + NTZ	38.3	**23.9**	5.0 *	**11.0 ^ns^**

^ns^ CefU + NTX + NTZ did not show statistical significance when compared to CefU + DSF + NTZ. * CefU + Clari/NTX + FZD and CefU + Clari + NTZ showed statistical significance when compared to CefU + DSF + FZD and CefU + DSF + NTZ, respectively. Abbreviations: untreated control (Cntrl), cefuroxime (CefU), disulfiram (DSF), clarithromycin (Clari), nitroxoline (NTX), furazolidone (FZD), nitazoxanide (NTZ).

**Table 4:** Subculture experiment (after 3 weeks) of a 7-day-old stationary phase culture after a 7-day treatment. Subcultures were performed in triplicate for the single drugs, as well as for the two-drug controls and three-drug combination of interest.

Drug	Subculture Results	Drug	Subculture Results
Cntrl	+++	FZD + Clari	+++
CefU	+++	FZD + NTX	+++
Clari	+++	NTZ + DSF	+++
DSF	+++	NTZ + Clari	+++
Doxy	+++	NTZ + NTX	+++
FZD	+++	NTX + DSF	+++
NTZ	+++	CefU + Doxy + Dapto	– – –
NTX	+++	CefU + DSF + FZD	+++
CefU + Doxy	+++	CefU + Clari + FZD	– – –
CefU + Clari	+++	CefU + NTX + FZD	– – –
CefU + DSF	+++	CefU + DSF + NTZ	+++
CefU + NTZ	+++	CefU + Clari + NTZ	– – –
CefU + NTX	+++	CefU + NTX + NTZ	– – –
FZD + DSF	+++	CefU + DSF + NTX	– – –

Each “+” (regrowth) or “–“ (no regrowth) symbol represents one replicate. Abbreviations: untreated control (Cntrl), cefuroxime (CefU), clarithromycin (Clari), daptomycin (Dapto), disulfiram (DSF), doxycycline (Doxy), furazolidone (FZD), nitazoxanide (NTZ), nitroxoline (NTX).

**Table 5:** Cmax concentrations for drug candidates. Cmax values presented in the table are for oral drugs, except for daptomycin, which is an intravenous antibiotic. When needed, Cmax was converted from a molar to a µg/mL concentration. For the purpose of this study, drugs were tested at their mean concentration value (range) ^a^.

Drug	Cmax Mean in µg/mL (Range)	Source
**Arte**	0.43	http://www.antimicrobe.org/ [39]
**CefU**	8.9 (4.2–13.6)	http://www.antimicrobe.org/ [40]
**Clari**	6.8	http://www.antimicrobe.org/ [41]
**CFZ**	1.1 (0.7–1.4)	Yawalkar and Vischer, 1979 [42]
**Cry**	0.007–0.024 ^b^	Forkuo et al., 2017 [33]
**Dapto**	77.5	http://www.antimicrobe.org/ [43]
**DSF**	0.4	Spillier et al., 2019 [44]
**Doxy**	2.6 (1.5–3.6)	http://www.antimicrobe.org/ [45]
**Ery**	3.0	http://www.antimicrobe.org/ [46]
**FZD**	0.3	Calafatti, Ortiz, Deguer, Martinez, and Pedrazzoli, 2001 [47]
**LNZ**	21.2 (15.4–27.0)	http://www.antimicrobe.org/ [48]
**NTZ**	10.6 (8.6–12.6)	Food and Drug Administration, 2005 [49]
**NTX**	5.6 (2.45–8.75)	Bergogne-Berezin, Berthelot, and Muller-Serieys, 1987 [50]
**Ribu**	0.4 (0.2–0.6)	http://www.antimicrobe.org/ [51]

^a^ When a range was available, the mean of the minimum and maximum Cmax values were chosen, except for Cry. In the specific case of Cry, we decided to take the upper limit of the Cmax range (0.024 µg/mL) as the concentration to evaluate in the drug exposure experiment at Cmax. ^b^ Cmax for cryptolepine was taken from a pharmacokinetic animal study with rodents, in which cryptolepine was orally administered at a dose of 5 mg/kg.

## Supplementary Materials

The following are available online at https://www.mdpi.com/2079-6382/9/9/542/s1, Figure S1: Effect of different drugs at 50 µM in a 7-day-old *B. burgdorferi* stationary-phase culture. Table S1: Susceptibility of *B. burgdorferi* in a 7-day-old stationary phase culture to 50 µM drugs after a 7-day treatment. Table S2: *B. burgdorferi* viability (in %) in a 7-day-old stationary phase culture after the drug exposure experiment at a 5 µg/mL concentration—two-drug combinations. Table S3: *B. burgdorferi* viability (in %) in a 7-day-old stationary phase culture after the drug exposure experiment for 7 days at a 5 µg/mL concentration per drug—three-drug combinations.
