# Supplementary material for: Evaluation of Disulfiram Drug Combinations and Identification of Other More Effective Combinations against Stationary Phase Borrelia burgdorferi
Source: Antibiotics (Basel). 2020 Aug 26;9(9):542. doi: 10.3390/antibiotics9090542 (PMC7559458; doi:10.3390/antibiotics9090542)
Supplement: Supplementary file 1 [file antibiotics-09-00542-s001.pdf]

Supplemental Information: *In-vitro* Evaluation of Disulfiram against Stationary Phase *B. burgdorferi*

Hector S Alvarez-Manzo, Yumin Zhang, Wanliang Shi and Ying Zhang

Table S1. Susceptibility of *B. burgdorferi* in a 7-day old stationary phase culture to 50μM drugs after a 7-day treatment. Survival rates were determined using the SYBR Green I/ PI assay.

Stationary phase culture (7-day old)		Stationary phase culture (7-day old)	
Drug (50 μM)	Survival rate (%)	Drug (50 μM)	Survival rate (%)
Untreated control (Cntrl)	88.9	Daptomycin (Dapto)	0.0
Cefuroxime (CefU)	35.4	Disulfiram (DSF)	39.4
Doxycycline (Doxy)	69.7	Erythromycin (Ery)	1.6
Amoxicillin (Amoxi)	46.0	Furazolidone (FZD)	0.7
Artemisinin (Arte)	4.4	Linezolid (LNZ)	1.1
Azithromycin (Azi)	2.6	Nitazoxanide (NTZ)	0.8
Clarithromycin (Clari)	14.3	Nitroxoline (NTX)	1.5
Clofazimine (CFZ)	2.0	Rifabutin (Ribu)	7.1
Cryptolepine (Cry)	1.3		

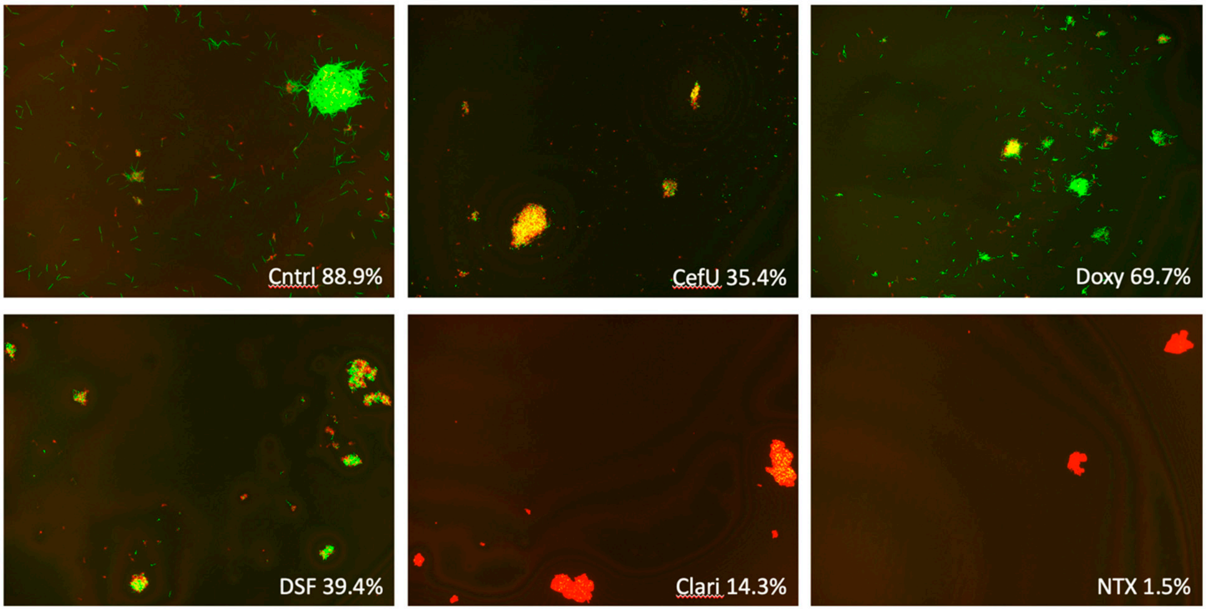

Figure S1. Effect of different drugs at 50 μM in a 7-day old *B. burgdorferi* stationary-phase culture. Abbreviations: Untreated control (Cntrl), Cefuroxime (CefU), Doxycycline (Doxy), Disulfiram (DSF), Nitazoxanide (NTZ), Nitroxoline (NTX)

Table S2. ***B. burgdorferi*** **viability (in %) in a 7-day old stationary phase culture after the drug-exposure experiment at a 5 µg/mL concentration.** Results in bold and underlined were further assessed in three-drug combinations (Table 5).

	Cntrl	CefU	Doxy	Amoxi	Arte	Azi	Clari	CFZ	Cry	DSF	Ery	FZD	LNZ	NTZ	NTX	Ribu
	87.7	36.0	52.6	49.9	66.1	47.4	42.1	47.9	32.1	34.1	49.2	64.1	61.4	70.7	32.6	64.6
CefU	—	—	**34.1**	—	37.6	36.8	24.6	36.7	21.2	32.4	23.1	27.5	34.5	29.2	24.0	31.2
DSF	—	**32.4**	32.4	**29.6**	**22.0**	36.7	**31.6**	**30.2**	**16.7**	—	**20.2**	**22.0**	37.1	**26.4**	**24.2**	**17.9**

A crossed line means values for these combinations were not determined. Abbreviations: Untreated control (Cntrl), Cefuroxime (CefU), Disulfiram (DSF), Doxycycline (Doxy), Amoxicillin (Amoxi), Artemisinin (Arte), Azithromycin (Azi), Clarithromycin (Clari), Clofazimine (CFZ), Cryptolepine (Cry), Erythromycin (Ery), Furazolidone (FZD), Linezolid (LNZ), Nitazoxanide (NTZ), Nitroxoline (NTX), Rifabutin (Ribu)

Table S3. ***B. burgdorferi*** **viability (in %) in a 7-day old stationary phase culture after the drug-exposure experiment for 7 days, at a 5 µg/mL concentration per drug: three-drug combinations.** Results in bold and underlined were further assessed at Cmax concentration (Table 5).

	Cntrl	CefU	DSF	Doxy	Arte	Clari	CFZ	Cry	FZD	LNZ	NTZ	NTX	Ribu
	87.7	36.0	34.1	52.6	66.1	42.1	47.9	32.1	64.1	61.4	70.7	32.6	64.6
CefU + Doxy	—	—	27.2	—	22.8	25.5	34.1	13.1	21.6	29.4	26.8	17.5	29.3
CefU + DSF	—	—	—	27.2	13.2	14.3	15.2	**1.4**	**9.1**	12.1	**10.1**	**8.3**	16.1
Cry + DSF	—	—	—	17.8	15.0	11.3	13.3	—	12.1	11.7	**7.3**	14.9	**9.0**
Ribu + DSF	—	—	—	23.8	18.1	16.1	**8.9**	**9.0**	13.6	15.6	**10.4**	22.4	—
Ery + DSF	—	—	—	19.0	24.3	—	21.7	**7.4**	26.2	22.3	19.6	21.3	18.3
Arte + DSF	—	—	—	22.9	—	12.0	21.8	15.0	23.8	13.8	16.5	20.1	18.1
FZD + DSF	—	—	—	27.9	23.8	18.3	**7.0**	12.1	—	**10.0**	**8.5**	**8.5**	13.6
NTX + DSF	—	—	—	17.7	20.1	**9.2**	17.8	14.9	**8.5**	**6.1**	12.8	—	27.2
NTZ + DSF	—	—	—	19.1	16.5	19.3	21.6	**7.3**	**8.5**	**9.5**	—	12.8	**10.4**
Amoxi + DSF	—	—	—	37.5	23.3	29.2	28.0	16.8	26.3	30.3	23.1	19.2	28.3
CFZ + DSF	—	—	—	31.9	21.8	19.0	—	13.3	**7.0**	19.1	21.6	17.8	**8.9**
Clari + DSF	—	—	—	24.4	12.0	—	19.0	11.3	18.3	**9.0**	19.3	**9.2**	16.1

A crossed line means values for these combinations were not determined. Abbreviations: Untreated control (Cntrl), Cefuroxime (CefU), Doxycyline (Doxy), Disulfiram (DSF), Artemisinin (Arte), Clarithromycin (Clari), Clofazimine (CFZ), Cryptolepine (Cry), Erythromycin (Ery), Furazolidone (FZD), Linezolid (LNZ), Nitazoxanide (NTZ), Nitroxoline (NTX), Rifabutin (Ribu)
